# Occurrence of Mycotoxins in Fish Feed and Its Effects: A Review

**DOI:** 10.3390/toxins12030160

**Published:** 2020-03-04

**Authors:** Mariana Oliveira, Vitor Vasconcelos

**Affiliations:** 1 Faculty of Sciences, Biology Department, University of Porto; Rua do Campo Alegre, Edifício FC4, 4169-007 Porto, Portugal; mariana.oliveira@ciimar.up.pt; 2 Interdiciplinary Center of Marine and Environmental Research (CIIMAR/CIMAR), University of Porto, Terminal de Cruzeiros do Porto de Leixões, 4450-208 Matosinhos, Portugal

**Keywords:** mycotoxins, mycotoxicosis, fungi, aquaculture, fish feed, fungi, bioaccumulation, toxicity

## Abstract

Plant-based ingredients have been successfully replacing fishmeal in finished fish feeds. However, using crops in feeds results in an increased risk of contamination by fungi and mycotoxins and a higher incidence of mycotoxicosis in fish. This might decrease aquaculture’s productivity as mycotoxicosis generally result in decreased body weight, growth impairment and higher rates of disease and mortality in fish. Additionally, some mycotoxins might accumulate in the fish musculature. As such, fish consumption might become another way for mycotoxins to enter the human food chain, threatening food security and public health as mycotoxins are important genotoxins, carcinogens and immunosuppressors to humans. In this work we aim to provide a review on the most important mycotoxins found in crops and in finished fish feed, i.e., aflatoxins, fumonisins, ochratoxins, trichothecenes and zearalenone. We present their effects on the health of fish and humans and their regulations in the European Union. Although work has been performed in mycotoxin research ever since the 1960s, a lot of information is still lacking regarding its effects. However, it is noticed that in order to use crops in aquafeed production, efforts should be made in order to monitor its contamination by mycotoxinogenic fungi and mycotoxins.

## 1. Introduction

Fish constitutes a source of proteins and other vital nutrients and, consequently, is a crucial component of the human diet. When compared to meat from terrestrial animals, fish has a higher content of protein and essential amino acids, long-chain omega-3 fatty acids, vitamins and most of the essential minerals and trace elements, particularly iodine, fluorine and trivalent chromium which0 1are usually lacking in other meat products [1]. Furthermore, several health benefits have been associated with modest fish consumption. In addition to other effects [2], it has been associated with a protective effect against cancer [3,4,5] and cardiovascular diseases [6,7,8], two of the main causes of death in the European Union countries [9].

In around 50 years, the worldwide per capita consumption of seafood (fish, crustaceans, mollusks and other aquatic animals except mammals) has more than doubled, turning from 9.0 kg in 1961 to 20.2 kg in 2015 [10]. Although various factors (e.g., price of fish, religion) may play a role in different countries, this increase was mostly attributed to the rapid population growth and income improvement in some developing countries, as well as changes in the dietary patterns due to the growing health and sustainability consciousness in developed countries [11]. The increase in fish consumption was also attributed to a boost in capture fisheries, but particularly in aquaculture production which occurred in the twentieth century. Aquaculture has, in fact, been growing and continues to grow faster than any other major food production sector, at an average annual rate of 5.8% (2000–2016) [10], allowing an increase in total fish supply, all while decreasing its market value [12].

Around 40.0% of fish produced by aquaculture for human consumption requires a high amount of externally provided protein-rich aquafeed. These are high-trophic carnivorous fish (e.g., salmon, tuna) which depend on externally provided feed to survive, but also non-obligate carnivore fish (e.g., carp) to which compound feed is being provided in order to boost growth and improve the farmer’s profits [13,14]. Commercial compound feed is a mixture of products of vegetable or animal origin in their natural state, fresh or preserved, or products derived from the industrial processing thereof, for oral feeding in the form of a complete feed, i.e., a nutritionally adequate formulated feed which solely provides the necessary nutrients to support the animal’s life [15]. In addition to sustaining life, aquafeed is supposed to produce the maximum growth and weight gain of the fish in the shortest period of time and maintain the health and the quality of the animal’s skin and musculature in order for it to then be consumed by humans. As such, it depends on highly nutritive ingredients such as fishmeal and fish oil.

Fishmeal constitutes a large portion of aquafeed as it is an important source of proteins and vital amino acids, among other micronutrients. Fish oil is a source of long-chain polyunsaturated fatty acids and it is produced as a by-product of fishmeal. Nearly 20.0 million tons of raw material is annually being used to produce around 4.6 million tons of fishmeal and 918.0 tons of fish oil [16]. Although these ingredients are used in other industries, aquaculture is estimated to have consumed almost 61.0% of world fishmeal production and 74.0% of fish oil production in 2008 [17]. Fish by-products are being more frequently used as raw materials in manufacturing, but 65.0% to 75.0% of fishmeal and fish oil are still being produced using whole fish, particularly wild-caught small pelagic forage fish such as anchovies, mackerel, sardines, sprat, menhaden and others [17]. As such, aquaculture still greatly depends on wild fish for the production of aquafeed and, although this dependency has been decreasing in recent years, a big part of capture fisheries is still being reduced into fishmeal and fish oil or used directly for animal feeding [18].

Aquaculture is expected to continue growing and will be a major food source in the future, contributing in the next decade to 62.0% of fish destined for human consumption [19]. Additionally, as more high-trophic level fish are being famed, even more fish needs to be caught in order to produce aquafeed, i.e., carnivorous fish produced by aquaculture require 2.5 to 5.0 times more fish biomass as feed input than the final weight attained by the fish which is being farmed [20]. To meet these new demands, the global production of aquafeed is projected to greatly increase in the near future [21,22]. However, demand for these fish might soon approach ecological supply due to, among other reasons, its use in aquafeed production [23]. The resulting shortage of pelagic fish might have negative ecologic and economic implications. Pelagic fish have a pivotal role in the ecosystems because they are a key part of various food webs, supporting many predators (fish, seabirds, marine mammals and squid) which are highly or extremely dependent on these fish [24]. In some regions of the globe, pelagic fish also are a part of the diets of humans. Sardines, for example, are eaten all around the world fresh, smoked, salted, dried or canned and are an important delicacy in countries like Portugal, Spain or Turkey. In addition to the negative impacts that the depletion of the wild stocks of pelagic fish would have on the biodiversity of marine ecosystems, it would also lead to less growth of wild species of edible fish and less catches of feed fish [25]. In turn, capture fisheries will suffer important impacts because pelagic fish contribute globally to this sector, directly or indirectly (by supporting its predators), to an estimate total of almost $17.0 billion USD [24]. A shortage of pelagic fish would also impact aquaculture production as the market price for these fish would rise and, consequently, so would the market prices for fishmeal and fish oil [19,26] and aquaculture production. 

Reducing aquaculture’s dependency on marine resources is necessary in order to make this industry economically and ecologically sustainable. This decrease will depend on substituting fishmeal and fish oil with viable, cost-effective and nutritionally similar alternatives. Several ingredients have been tested to replace fishmeal and fish oil (e.g., microalgae, bacteria, etc.) in both farm-made and industrialized aquafeed and the most common alternatives have been terrestrial crops. Plant oils, such as palm, peanut or rapeseed oils, have been increasingly used to replace fish oil, but they have a low concentrations of long-chain omega-3 fatty acids and cannot be used to completely replace this ingredient (although they are used in blending rations) [27]. Currently, there are no viable alternatives for fish oil. Fishmeal, however, has been successfully replaced in commercially and farm-made aquafeeds by plant-based materials like oil seeds, legumes and cereal grains such as barley, canola, corn, cottonseed, peas/lupins, and wheat. Currently, these represent the biggest dietary source of protein for lower trophic level fish species (tilapias, carps, catfishes) and the second major source of protein for shrimp and European high trophic level fish species [21].

Soy products are the most promising substitutes for fishmeal because they are widely available. Soybean meal, produced as a by-product of processing mature soybeans (*Glycine max*), is the most common plant-based ingredient used in fish feed and usually represents 15.0% to 45.0% of aquafeed for herbivorous and omnivorous fish [21]. Regarding carnivorous, high-trophic level fish, plant protein concentrates appear to be the solution to replace fishmeal. For example, soy protein concentrate, another by-product of soybeans’ processing, is nutritionally similar to fishmeal—it has a high crude protein content (65% to 67%) and a balanced amino acid level—and has high digestibility and palatability [28]. Indeed, it has been showed that 40.0% to 100.0% of fishmeal could be replaced by soy protein concentrate without negative effects on the growth performance of different species of fish [29]. 

The main disadvantage of using crop ingredients in fish feed is that they have a high composition of anti-nutritional factors. These antinutrients (e.g., trypsin inhibitors, saponins, etc.) adversely affect the digestive as well as metabolic activity of the animal, leading to diverse negative effects on its health [30,31]. Additionally, mycotoxins are also frequently identified in plants. Mycotoxins are naturally occurring molecules produced as secondary metabolites by filamentous fungi, most commonly by species of the genera *Aspergillus*, *Penicillium* and *Fusarium*, in a strain-specific way [32,33]. Twenty-five percent of global food crops are contaminated by these toxins, although the number varies widely depending on many factors (e.g., commodity analyzed, mycotoxin studied, etc.) and can actually reach 60.0% to 80.0% for some mycotoxins [34,35].

Toxinogenic fungi can be categorized into two groups: field fungi (e.g., *Fusarium* spp.), which gets access to the crop during the development of the plant, or storage fungi (e.g., *Aspergillus* spp., *Penicillium* spp.) [30], which mostly contaminate the crop post-harvest. The detection of these fungi in feed or its raw materials does not necessarily mean that they will be contaminated by mycotoxins. Several factors such as the strain which is detected, substrate composition, moisture content, aeration, temperatures and other storage conditions affect the production of these toxic metabolites, although generally hot and humid conditions are the two main factors leading to fungal growth and toxin production [36]. 

Contamination by mycotoxins can result in deterioration and reduction in the nutritional value of the ingredients and/or the aquafeed produced, but also might pose an important health risk for both fish and humans. When mycotoxins are ingested by fish, they might not only affect the animal’s wellbeing, but might also be passed through the food chain to its consumer and lead to serious health effects. Mycotoxicosis are intoxications which occur in animals and humans as a consequence of the intake into the organism of one or more mycotoxin [37] and which can result in disease or death. The main health burden of mycotoxin exposure is related to its chronic toxicity [33]. Chronic mycotoxicosis leads to adverse effects that are manifested after long-lasting exposure to a low dose of mycotoxin (e.g., cancer induction, impaired growth, immune dysfunction, etc.), while acute mycotoxicosis is manifested rapidly following exposure to a large amount of mycotoxin [38]. The symptoms of a mycotoxicosis depend on the type of mycotoxin, the amount and duration of exposure, and the age, health and sex of the exposed individual, among other issues involving genetics, dietary status and interaction with other toxic compounds [33].

## 2. Mycotoxin Contamination of Fish Feed

Mycotoxin contamination of crops might occur pre-harvest, particularly in agriculture commodities which are bran- or fiber-enriched and which have high mold and high moisture content. Contamination can also occur post-harvest or during storage in inappropriate conditions which will favor mycotoxin production, i.e., when temperature and water activity increases to levels which will allow the optimal conditions for fungal growth and mycotoxin production [36]. Once an ingredient or finished feed is contaminated, there are currently no methodologies available to eliminate mycotoxins. However, different processing methods might help in reducing mycotoxin concentrations, particularly those which use higher temperatures [39].

The trend to use plant-based materials in aquafeeds appears to be increasing [23]. However, contamination of these ingredients with potentially mycotoxinogenic fungi, particularly *Aspergillus flavus* and *Aspergillus parasiticus*, is frequent and occurs virtually everywhere [40]. In Serbia, zearalenone, ochratoxin A and aflatoxin B_1_ were detected in corn, wheat and barley destined for fish feed production. The authors recorded particularly high levels of zearalenone in corn (mean of 5.3 mg/kg) [41]. In Brazil, aflatoxin B_1_ was detected, although in low doses (1.1 µg/kg to 7.4 µg/kg), in samples of soybean bran, corn bran and other cereals from fish farms [42]. Aflatoxin B_1_ (1.0 to 135.0 µg/kg), together with fumonisins (261.0 to 2420.0 µg/kg), were also detected with high incidence in Malaysia, in corn samples intended for feed manufacture [43]. In Portugal, aflatoxin B_1_ was detected in a range of concentrations between 1.0 and 45.0 µg/kg in all samples of feedstuff, i.e., soy, rice, corn, wheat and barley analyzed [44]. Additionally, the authors found deoxynivalenol (100.0 to 500.0 µg/kg) and fumonisin B_1_ (10 to 40 µg/kg) in rice, corn, wheat and barley [44]. 

The mycotoxins described above which were detected in feed ingredients have all been previously detected in finished aquafeed as well. In Brazil, aflatoxin B_1_ was detected at a mean concentration of 3.8 µg/kg (from 1.6 to 9.8 µg/kg) [42]. It was also detected at mean levels of 1.40 µg/g, together with fumonisins (mean, 1.60 µg/g), in commercial feeds [45]. In this country, there was also a high incidence of fumonisin B_1_ in locally produced finished feed intended for Nile tilapia, in mean levels of 2.6 µg/g. Aflatoxin B_1_ and ochratoxin A were also detected [46]. In Nigeria, aflatoxin B_1_ was detected in 92.0% of locally formulated fish feed in levels of up to 550.8 µg/g [47]. This toxin was also found in 23.3% of fish feed samples analyzed in Egypt, together with ochratoxin A [48]. In Bagdad, deoxynivalenol was detected at a concentration range from 2.0 to 5.0 mg/kg, while zearalenone was detected at concentrations of 0.5–5.0 mg/kg [49]. In Kenya, aflatoxins were detected in 84.0% of fish feed samples, in a mean concentration of 7.0 µg/kg (1.8 to 39.7 µg/kg) [50]. It is important to notice that mycotoxins are more commonly found and in higher concentrations in farm-made feed than in commercial feed [50]. As farm-made feed is more commonly formulated in developing countries, this might contribute to why contamination by mycotoxins is more frequent developing countries.

Overall, the contamination of fish feed with mycotoxins appears to be common and a worldwide issue, but the type and prevalence of mycotoxin contamination in feed appears to highly depend on the geographical region. Aflatoxins are most often detected in Southern Europe, Africa, South Asia and Southeast Asia, deoxynivalenol is more frequent in North America, Northern and Central Europe, Africa and North Asia, zearalenone contamination has a higher incidence in North and South America, Central Europe, Africa and North and Southeast Asia, higher incidence of fumonisin was seen in South America, Southern Europe, Africa, North, South and Southeast Asia and ochratoxin A was most prevalent in South Asia and Africa (reviewed by Pinotti et al., 2016) [51]. According to these authors, multi-toxin contamination was more prevalent in Asia than in Europe or America. These differences might be due to the climate of each country, the type of samples analyzed, or the methodology used to identify mycotoxins, among other factors.

Considering the high frequency of mold and mycotoxin contamination in crops, and because mycotoxin contamination will become more prominent due to climate change [52,53,54], it is important to question whether food security, but also public health, are at risk. With this in mind, this review provides information on the most important mycotoxins found in fish feed and its raw materials—aflatoxins, fumonisins, ochratoxins, trichothecenes and zearalenone, in no particular order—and their effects on the health of fish and humans. Although for this review masked mycotoxins are not considered, it is important to note their existence as they are also important contaminants of plant-based ingredients. Masked mycotoxins are mycotoxin derivates that are undetectable by conventional analytical techniques (e.g., high-performance liquid chromatography or some enzyme-linked immunosorbent assays) because their chemical structure has been altered by plant enzymes involved in their defense mechanisms against xenobiotics [55]. Although data regarding their toxic effects are scarce, several studies highlight their potential threat to consumer safety [56,57,58].

## 3. Aflatoxins and Its Precursors

Aflatoxins were the first mycotoxins to be discovered, after a case of what was later found to be acute aflatoxicosis (“Turkey X disease”) resulted in the death of around 100,000 turkeys in the 1960s [59,60]. Currently, out of all mycotoxins, aflatoxins are the most studied and best characterized.

The most important aflatoxins in crops are aflatoxins B_1_, B_2_, G_1_, and G_2_. These toxins are mainly produced by *A. flavus* (only B-type aflatoxins), but *A. parasiticus* and, more rarely, *Aspergillus nomius* can also synthesize them. Other filamentous fungi of the genera *Penicillium*, *Rhizopus*, *Mucor* and *Streptomyces* are also producers of aflatoxins [61]. The biosynthesis of aflatoxin B_1_ requires sterigmatocystin, which is its precursor. Sterigmatocystin is mainly produced by *Aspergillus versicolor* and *Aspergillus nidulans*, but synthesis can also occur by other *Aspergillus* spp. (sections *Ochraceorosei*, *Nidulantes*, *Flavi* and *Versicolores*) or other filamentous fungi [62].

A wide variety of feedstuffs (maize, corn, wheat, cottonseed, nuts, among others) [63] might become contaminated with aflatoxins. However, the main sources of these toxins in animal feeds are groundnut meal, maize and cottonseed meal [64]. Regarding sterigmatocystin, it has been found to contaminate agriculture commodities used as feed ingredients such as wheat, corn, maize, barley or soybean [65,66]. Contamination can occur both pre- and post-harvest and is particularly frequent if plants are under stress, i.e., during high heat and drought or after insect infestation. After contamination with aflatoxin-producing fungi, improper storage might lead to the production of aflatoxins. Synthesis of these fungal metabolites is increased when feed is subjected to temperatures above 27.0 °C, humidity levels above 62.0% and moisture levels in the feed greater than 14.0% [67].

Regulations regarding the maximum levels of mycotoxins which can be present in foodstuff and feed have been set in different countries [61]. There are significant differences between these values, mainly due to different dietary patterns and, consequently, different intake of crops. The regulations set by the European Commission, however, appear to be the most cited. According to them, all cereals and derived products should have less than 2.0 μg/kg of aflatoxin B_1_, while in maize and rice this limit cannot exceed 5.0 μg/kg. As for the sum of aflatoxins B_1_, B_2_, G_1_ and G_2_, all cereals and its derived products should not have a concentration of 4.0 μg/kg or above, while for processed maize and rice the limit is 10.0 μg/kg [68]. The maximum level of aflatoxin B_1_ in all feed materials is 0.02 mg/kg and in complete feedstuff (other than to feed cattle, sheep, goats, dairy animals, calves, lambs, pigs and poultry, i.e., fish feed) it is 0.01 mg/kg [69]. No regulations have been set for sterigmatocystin.

Aflatoxin B_1_ is the most hepatocarcinogenic natural substance known to man. Apart from being a carcinogen, it also has genotoxic and immunodulatory effects on animals and humans. Overall, exposure of aflatoxin B_1_ results in poor growth, anemia, impaired blood clotting, sensitivity to bruising, damage to the liver and other organs, decreased immune responsiveness resulting in an increased vulnerability to bacteria, viral or parasitic infections, and increased mortality [40,70]. The in vivo effects on fish of aflatoxins and sterigmatocystin are described in Table 1.

### 3.1. Effects on Fish

Clinical signs associated with aflatoxicosis in fish include pale gills, impaired blood clotting, poor growth rates and lack of weight gain [61]. Visible signs of severe infection might be reduced survival rate, darkening/yellowing of the body and abnormal behavior, as seen in Nile tilapia and juvenile sturgeon [40,71,72].

Fish’s susceptibility to aflatoxins depends on the age and species, i.e., fry are more susceptible than older fish and some species are more sensitive than others [67]. Rainbow trout (*Oncorhynchus mykiss*) is the most sensitive fish species to aflatoxins. Chronic exposure to low levels of this toxin (as low as 0.0004 mg/kg) has been shown to increase the chances of rainbow trout developing cancer [50,67]. When these fish were fed a diet with 0.02 mg/kg of aflatoxin B_1_ for 8 months and for 12 months, the occurrence of liver tumors was 58.0% and 83.0%, respectively [67]. In addition to cancer, in vitro exposure to aflatoxin B_1_ drives to dramatic immunologic suppression in trout [73]. It was determined that the lethal dose capable of killing 50% of the analyzed rainbow trout population (LD_50_) was 0.81 mg/kg of bodyweight for aflatoxin B_1_ [74]. Sea bass (*Dicentrarchus labrax L*.), for which the acute oral lethal concentration (96 h) to aflatoxin B_1_ was as low as 0.18 mg/kg body weight [72], and red drum (*Sciaenops ocellatus*), who were affected by 0.0001 mg/kg of aflatoxin B_1_ [75], are other fish species which are highly sensitive to aflatoxin B_1_.

Warm water fish are reported to be less sensitive to aflatoxins than freshwater fish [70]. The LD_50_ calculated for channel catfish (*Ictalurus punctatus*) was 11.5 mg/kg of bodyweight (intraperitoneal administration) [76], around ten times higher than that of rainbow trout. Nile tilapia (*Oreochromis niloticus*) has also been shown to be less sensitive than rainbow trout to aflatoxin B_1_ [50]. The LD_50_ calculated for Nile tilapia was 1.0 and 1.3 mg/kg of bodyweight to aflatoxin B_1_ [77]. However, the intake of up to 3.0 mg/kg of aflatoxin B_1_ seems to not affect the survival rate of these fish [40,78,79,80]. The effects of aflatoxin B_1_ are, however, dose- and duration-dependent [78].

Exposure to sterigmacystin causes similar health effects as exposure to aflatoxins, i.e., it is also a genotoxin and a carcinogen which mostly affects the liver and kidneys. However, sterigmatocystin is less toxic than aflatoxin [65,81]. Exposure to sterigmatocystin has been shown to cause hepatocellular carcinoma in rainbow trout [82] and DNA damage, chromosomal aberration in the kidneys, increased frequency of micronucleate red blood cells and histopathological lesions of the liver, spleen and gills of Nile tilapia [83,84]. In addition, a relatively high mortality rate after sterigmatocystin intoxication was noted in these studies.

Bioaccumulation of aflatoxins and sterigmatocystin has been proven to occur in different fish species. Aflatoxin B_1_ residues have been detected, although in low doses, in the hepatopancreas, the ovaries and muscle of gabel carp (*Carassius auratus gibelio*) [85,86], in the liver and muscle of lambari fish (*Astyanax altiparanae*) [87] and in the muscle of sea bass (approx.: 0.005 mg/kg) [72]. Regarding sterigmatocystin, Abdel-Wahhab et al. (2005) [83] detected the presence of this toxin’s residues in Nile tilapia’s edible tissues (around 8 µg/kg tissue). Although accumulation in the fish’s musculature might occur, sterigmacystin has been showed to be of low concern for public health [65].

### 3.2. Effects on Humans

The main source of human exposure to aflatoxins is the ingestion of contaminated food, with the burden of dietary exposure being particularly high in developing countries. The mean aflatoxin exposure is estimated to be less than 1.0 ng/kg of body weight per day in developed countries, whereas in sub-Saharan African countries it exceeds 100.0 ng/kg of bodyweight per day [88]. Exposure might be through the direct ingestion of this toxin, i.e., by eating crops contaminated by aflatoxins. Exposure can also be indirectly by consuming animal protein (e.g., fish) in which aflatoxin residues accumulated in the muscle after they were fed with feed contaminated by mycotoxins.

Aflatoxin B_1_ has potent genotoxic and carcinogenic effects. It has been classified as a group-1 carcinogen by the International Agency for Research on Cancer (IARC) of the World Health Organization [89], being particularly toxic to individuals who are infected by the hepatitis B virus [64]. Chronic exposure to aflatoxins has been associated with 28.0% of hepatocellular carcinoma globally, but the percentage of cases attributable to these toxins ranges from 0.0% in Europe and North America to 40.0% in Africa [90]. Acute exposure, on the other hand, results in severe damage to the liver and a high mortality rate. Acute aflatoxicosis has been described ever since the 1960s [61] and the most recent episodes occurred in eastern Kenya (2004) [91] and central Tanzania (2016) [92]. Together, these cases of acute aflatoxicosis affected 385 people, out of which 145 died. The signs of aflatoxicosis shown by these patients were jaundice, abdominal pain, vomiting, diarrhea and ascites. The apparent cause of both acute aflatoxicosis cases appears to be the ingestion of maize contaminated by aflatoxins.

Sterigmacystin is a possible human carcinogen (group-2B carcinogen) [89]. It has hepatotoxic effects after oral administration to different mammals. There is a lack of information regarding the effects of sterigmacystin on human health. More studies regarding exposure data, occurrence and toxicity are warranted in order to correctly identify its risk to consumers [66].

## 4. Fumonisins

Fumonisin B_1_ is the most toxic fumonisin. It is synthesized by several *Fusarium* species, among which *Fusarium verticillioides* is the frequent producer, but also by *Fusarium proleferatum* and *Fusarium nygamai*. *Alternaria alternata* is also a producer of fumonisin B_1_. Contamination by fumonisin occurs mostly in maize and its by-products, with this toxin being detected in 80% to 100% of corn samples in Mozambique, Burkina Faso, China and Malaysia [43,94,95].

Regulations for the maximum limits of fumonisins in cereals have been described exclusively for co-contamination with fumonisin B_1_ and B_2_. The maximum level allowed in unprocessed maize (with the exception of unprocessed maize intended to be processed by wet milling) is 4000.0 μg/kg. In feedstuff, fumonisins should not exceed 60.0 mg/kg in maize and maize products, while in complete feed for fish they should not exceed 10.0 mg/kg [96].

### 4.1. Effects on Fish

The results of several studies which have been performed regarding the effects of fumonisins on fish are summarized on Table 2. Susceptibility to fumonisins appears to be species-specific, just like it is for aflatoxins. Channel catfish appear to be moderately susceptible. Exposure of this fish to fumonisin B_1_ resulted in histological changes in the liver, growth suppression and low survival when high doses of this toxin (>320 mg/kg) were tested. The low survival rate observed when high concentrations of fumonisins were administered was caused by *Cytophaga* infection [97], possibly indicating an impairment in the immune system. Yieldrim et al. (2007) also determined that concentrations of 20.0 mg/kg or more of fumonisin reduces growth rate, but it also leads to an increase in free sphinganine to free sphingosine ratio [98], possibly impacting the sphingolipids metabolism and potentially leading to cell death or cell proliferation. However, Brown et al. (1994) described no relevant effects other than reduced weight gain when catfish were fed a diet containing 313.0 mg/kg of fumonisins for five weeks [99]. As such, conclusions about the effects of fumonisin on catfish are difficult to ascertain.

Exposure of Nile tilapia to fumonisins resulted in similar effects to those described by Yieldrim et al. (2007), i.e., reduction in growth rate and changes in the sphingolipids metabolism [100]. As such, these fish also appear to be moderately sensitive to fumonisins. Common carp (*Cyprinus carpio*), however, appear to be highly susceptible to damages produced by fumonisins. Exposure of these animals to 10.0 mg/kg and 100.0 mg/kg of fumonisins B_1_ for 42 days injured a wide variety of organs, such as the liver, pancreas, kidney, heart and brain, and led to blood vessel damages [101]. In the brain in particular, fumonisin B_1_ led to histopathological changes and vasculature of young carp [102].

Overall, fumonisins appear to induce organ damage, impairment of the immune system, reduction in weight gain, metabolic alterations which can result in cancer and increased mortality. There were no reports regarding the accumulation of fumonisins in the musculature of fish. As such, fish consumption does not seem to implicate any food security risk regarding this toxin. However, more studies are necessary to understand the impacts of these toxins on fish.

### 4.2. Effects on Humans

Fumonisins appear to only be dangerous to humans when they are chronically exposed to this toxin. Fumonisins B_1_ are a group-2B carcinogen [89] and, as such, are cancer-promoting toxins. They have been associated with a higher incidence of esophageal and hepatic cancer in China [95,103,104] and in Africa [105], in regions where contamination by fumonisins is highly frequent. Additionally, exposure to fumonisins during pregnancy appears to be related to a higher neural tube deformity risk in offspring [106].

## 5. Ochratoxin

The most toxic of the ochratoxins is ochratoxin A, which is produced by *Penicillium* spp. (*Penicillium verrucosum*) and *Aspergillus* spp., mainly by *Aspergillus ochraceous* and *Aspergillus carbonarius*. Contamination occurs most frequently post-harvest in cereal grains (wheat, barley, oats or corn), although it also happens in other commodities [63]. Ochratoxin A is particularly stable and, consequently, extremely difficult to eliminate which, together with its long half-life, makes it easy to be transported along the food chain.

The maximum level of ochratoxin A established by the European Commission is 5.0 μg/kg for unprocessed cereals and 3.0 μg/kg for all products derived from unprocessed cereals, including processed cereal products [68]. Cereal and cereal products used in feedstuff should not exceed 0.25 mg/kg of ochratoxin A [96]. There are no limits regarding complete feedstuff for animals other than pigs and poultry, possibly because the effects of these toxins are still widely unknown.

### 5.1. Effects on Fish

Studies regarding the effects of ochratoxin A on fish are scarce, but fish appear to be particularly sensitive to this mycotoxin and its effects on several fish species are described onTable 3. Adult sea bass (*D. labrax L*.) in particular are highly sensitive to ochratoxin A. The acute oral LC_50_ (96 h) was determined to be 277.0 μg/kg, with clinical signs of intoxication being nervous and respiratory manifestations [107]. Juvenile catfish suffered a significant reduction in body weight gain only two weeks and at each successive weighting after exposure to ochratoxin A (2.0 mg/kg and above). Feed conversion and hematocrit were also reduced, while the mortality rate among fish fed with 8.0 mg/kg of ochratoxin A increased and lesions on the kidney and liver were seen [108]. Additionally, juvenile channel catfish also suffered a higher mortality rate after being exposed to ochratoxin A (4 mg/kg) and *Edwardsiella ictalurid,* a bacteria which infects a variety of fish species and can cause septicemia and encephalitis [109]. This probably resulted from a decrease in immune response caused by ochratoxin A. In Nile tilapia, ochratoxin intoxication resulted in sluggish swimming, refusal to eat, reduced survivability, decreased growth performance, degenerative lesions in the kidney and liver consistent with necrosis, and reduction in total protein, among other effects [110].

The accumulation of ochratoxin in the muscle of fish does not appear to occur in rainbow trout [111] and Nile tilapia [112], and only low levels (mean 0.12 µg/kg) of ochratoxin A were found in the muscle of European seabass and gilthead seabream [112]. As such, fish might contribute to the presence of ochratoxin A in the food chain.

### 5.2. Effects on Humans

Studies regarding the effects of ochratoxin A on humans are scarce and thus they are widely unknown. However, it has been considered a group-2A carcinogen, i.e., a probable carcinogen [89]. It affects mainly the kidney, liver and blood, where it accumulates [113]. Ochratoxin A has genotoxic effects which result in DNA damage which, in turn, is the first step to carcinogenesis [114]. As such, exposure to this toxin may be involved in the development of hepatic cancer, urinary tract tumors and testicular cancer, among other diseases which have been widely reviewed by Malir et al. (2016) [115]. Ochratoxin A also seems to accumulate with high incidence, particularly in developing countries, in the breast milk of lactating women [113,116], which might lead to infant exposure.

## 6. Trichothecenes

Trichothecenes are produced in crops such as corn, wheat, barley and oats [63] by fungi of the genera *Fusarium*, *Myrothecium*, *Phomopsis*, *Stachybotrys*, *Trichoderma*, *Trichothecium*, among others [33]. The two most important trichothecenes found in crops, i.e., the most toxic to animals, are deoxynivalenol and T-2 toxin. Although exposure to T-2 toxin had important effects on the health of zebrafish embryos (increasing mortality and malformation, cardiovascular defects and behavioral changes) [118], this toxin does not seem to be a threat to the health of the fish [119,120]. However, this is not the case for deoxynivalenol (also called vomitoxin) which is the least toxic trichothecene, but can cause harm to fish and humans. Deoxynivalenol is mostly produced by *Fusarium* spp., most notably by *Fusarium graminearum*, and appears to be more common in feed when compared to food in Europe [121].

The maximum level of deoxynivalenol allowed by the European Commission in foodstuff is 1750.0 μg/kg for unprocessed durum wheat, oats and maize (with the exception of unprocessed maize intended to be processed by wet milling) and 1250.0 μg/kg for any other unprocessed cereal [68]. As for feed materials, the maximum level of deoxynivalenol in maize by-products is 12.0 mg/kg and 8.0 mg/kg for other cereals and cereal products. In complete and complementary feedstuff (except for pigs, calves, lambs and kids), deoxynivalenol should not exceed 5.0 mg/kg [96].

### 6.1. Effects on Fish

The effects of deoxynivalenol on fish are still widely unknown. However, in vitro tests identified fish as sensitive animals to this toxin (Table 4). Rainbow trout, in particular, appears to be the most sensitive fish species [122]. Generally, exposure of this fish to deoxynivalenol does not result in higher mortality. However, when doses of up to 2.6 mg/kg of this toxin were fed to rainbow trout, it resulted in feed refusal and reduction in feed conversion efficiency which, in turn, led to a reduction in weight gain and growth rate [123,124,125,126]. However, feeding rainbow trout a diet with 6.4 mg/kg of deoxynivalenol resulted in a reduction in mortality after *Flavobacterium psychrophilum* infection, although in parallel there was an increase in feed refusal [126,127]. Similarly, exposure of channel catfish to deoxynivalenol (2.5 to 10.0 mg/kg) increased their survival rate after *Edwardsiella ictalurid* infection, but with no negative effects on weight gain and feed conversion efficiency [128]. Thus, deoxynivalenol appears to have a protective effect against bacterial infections in some species of fish.

Juvenile Atlantic salmon (*Salmo salar*) are fairly susceptible to deoxynivalenol intoxication. When these fish are exposed to deoxynivalenol (4.0 and 6.0 mg/kg), and just as it happens with rainbow trout, there was a reduction in feed intake, in weight gain and in feed efficiency. Additionally, there was a reduction in plasma protein (total protein and albumin) and lipids (triglycerides and cholesterol), which might result from a reduction in protein and lipoprotein synthesis in the liver [117]. Changes in protein expression also appear to impair the intestinal integrity of Atlantic salmon which affects both the structure and function of its intestine [129].

No effects on growth or mass of carp were observed when they were exposed for six weeks to doses of 352.0, 619.0 and 953.0 µg/kg of deoxynivalenol. However, immunosuppressive impairment was noted after exposure, as well as changes in blood hematology (increase in red blood cell formation) and pro-inflammatory immune reaction, although the meaning of this change is unknown [130].

Although deoxynivalenol has been found to accumulate in the muscle of carps [130] and rainbow trout [126], it only did so at low levels. In fact, deoxynivalenol is usually rapidly metabolized and generally does not accumulate in the animal’s organs.

### 6.2. Effects on Humans

Deoxynivalenol produces its toxic effects by inhibiting protein synthesis. It does not pose a health threat to humans compared to other mycotoxins as its effects are generally gastrointestinal, i.e., short-term nausea and vomiting, diarrhea, abdominal pain, headache, dizziness and fever [131]. In fact, trichothecenes in general, but particularly deoxynivalenol, have been associated with an outbreak of acute mycotoxicosis which occurred in India after consumption of bread made using mold-damaged wheat and led to severe gastrointestinal problems [132].

## 7. Zearalenone

Zearalenone is mostly produced by *Fusarium* spp., particularly *F. graminearum*, but also *Fusarium culmorum*, *Fusarium equiseti* and *Fusarium. crookwellense* [33]. Contamination by these fungi occurs mostly pre-harvest in crops such as corn [63]. Zearalenone has a strong estrogenic activity, i.e., is a mycoestrogen which affects the reproductivity ability of a variety of animals (as reviewed by Zhang et al., 2018) [133].

The European Commission has established a maximum level of zearalenone in foodstuffs of 350.0 μg/kg for unprocessed maize (with the exception of unprocessed maize used to be processed by wet milling) and 100.0 μg/kg for other unprocessed cereals [68]. In feeds, maize byproducts should not exceed 3.0 mg/kg of deoxynivalenol, while for other cereals and cereal products this limit is 2.0 mg/kg [96]. There are no limits established for complete feedstuffs for animals other than mammals.

### 7.1. Effects on Fish

Zearalenone accumulated in high amounts in the ovaries of rainbow trout (up to 7.1 µg/kg) [134], but the effects of this accumulation on reproduction as still widely unknown. However, short-time exposure of zebrafish (*Danio rerio*) to zearalenone was shown to decrease its reproductive ability by reducing spawning frequency [135] and relative fecundity between generations [136] (Table 5). Apart from its reproductive effects, zearalenone (2.0 mg/kg) appears to increase growth rate and feeding efficiency of rainbow trout, albeit its immunodulatory effects that might have perturbed the fishes’ health [137].

Apart from accumulating in the ovaries of rainbow trout, trace amounts of this toxin were also detected in the liver and intestines (<2.0 µg/kg) [134]. Zearalenone does not seem to accumulate, however, in the musculature of fish and, as such, fish consumption does not seem to be a source of this mycotoxin.

### 7.2. Effects on Humans

As zearalenone resembles the chemical structure of naturally occurring estrogen, it can bind to its receptors on the human cell leading to hormonal imbalances. In turn, this can result in a number of pathologies of the reproductive system. For example, zearalenone has been detected in hyperplastic and neoplastic endometrium [138], possibly contributing to carcinogenesis. However, according to IARC, zearalenone is not classifiable regarding its carcinogenicity to humans, i.e., there is no evidence that it causes cancer in humans [89]. Thus, more studies are needed in order to fully elucidate the effects of zearalenone on human health.

## 8. Co-Contamination by Different Mycotoxins

Multi-toxin contamination of fish feed might result from the contamination of the commodities used to produce compound aquafeed by fungi which produce more than one mycotoxin simultaneously. This might occur with *Fusarium* spp. which can produce more than one toxin in the crops which it infects. For example, contamination with zearalenone and fumonisins [139] and contamination with fumonisin and trichothecenes [104] have been described in maize. Co-contamination of crops and feed might also result from the contamination by different fungi which synthetize different mycotoxins. Sorghum, for example, was found to be contaminated by both *Fusarium* and *Aspergillus* and, consequently, by fumonisin B_1_ and aflatoxin B_1_ after storage for a long period of time [140]. Co-contamination of fish feed with mycotoxins has also been described [46,141].

The effects of ingestion of feed and food concurrently contaminated by different mycotoxins are still widely unknown, particularly the effects on fish, and more in vitro and in vivo studies are necessary to understand their biological activity. However, after being consumed, different mycotoxins might interact between each other [142] and, consequently, have synergetic and/or additive effects, particularly if the mechanisms of action are similar. Ochratoxin A and citrinin, for example, are two nephrotoxic mycotoxins which together exert synergic effects; the consumption of multiple trichothecenes, which are immunosuppressors, results in additive effects in a wide variety of animals [143]. Thus, they might impose a particularly elevated threat to the consumption of fish contaminated by mycotoxins.

## 9. Conclusions

The use of plant-based ingredients in aquafeed might impose serious threats to aquaculture’s productivity through an increased rate of mycotoxin contamination. Intake of mycotoxins by fish increases the disease and mortality rates and the incidence of reproductive issues and reduces weight gain, leading to important economic losses. In addition, the accumulation of even small doses of mycotoxins in the fish’s musculature might impose a serious health threat to its consumers. On one hand, this contributes to the already high burden of exposure to these toxic metabolites, particularly in developing countries or in regions where cereal consumption is high. On the other hand, human exposure to small doses of mycotoxins over a long period of time might result in chronic effects such as cancer or immunodeficiency. Thus, the presence of mycotoxins in aquafeed has severe impacts in both the economy and in public health. As such, strategies to control contamination, both pre- and post-harvest, and decrease exposure are fundamental. Monitoring of the raw ingredients as well as the finished feed should become a common practice to safeguard aquaculture as it grows in the future.

## Figures and Tables

**Table 1:** In vivo effects of aflatoxins and sterigmatocystin on fish. Effects were dose dependent.

Type of Mycotoxin	Fish	Species	Dose (mg/kg)	Exposure Time	Effects	Ref.
Aflatoxin B_1_	Juvenile 18-month sturgeon	*Huso huso*	0.01	<15 days	Decreased feed intake and weigh loss. Increased weakness in the performance. Changes in swimming behavior. Hemorrhagic skin lesions in the head and abdomen. Yellow spots in the pectoral area. Curvature of the spinal cord. Accumulation of exudes liquid in the ventricular and kidney. Hepatitis and liver cancer. Hyperinflammation of gallbladder.	[71]
Aflatoxin B_1_	Adult sea bass	*Dicentrarchus labrax L.*	0.018	42 days	Abnormal behavior—sluggish movements, swimming imbalance, rapid opercular movement and loss of equilibrium. Muscular seizures prior to death. Hemorrhages and yellow patches in the dorsal skin surface. Ascites. Hemorrhagic fluid in the abdominal cavity. Darkening of the body surface. Internal generalized congestion and pale discoloration of liver, kidney and gills. Severe distention of the gallbladder. Changes in eye opacity and exophthalmia. Increase in serum transaminases and alkaline phosphatase activities. Decrease in plasma proteins, albumin and globulin. Emaciation. Residual high levels (≈0.005 mg/kg) in the musculature.	[72]
Aflatoxin B_1_	Channel catfish	*Ictalurus punctus*	12	10 days	Regurgitation of the stomach contents. Discoloration of the gills, livers, kidneys, spleen, stomach and intestines. Reduction in hematocrits, hemoglobin concentrations and erythrocyte counts. Histological lesions in the intestinal mucosa. Necrosis of hematopoietic tissues, hepatocytes, pancreatic acinar cells and gastric glands. Reduction in the volume of red pulp and the number of leukocytes in the spleen. Dilation of the renal tubular lumens.	[76]
Aflatoxin B_1_	Channel catfish	*Ictalurus punctus*	2.2–10	10 weeks	Reduced growth rate. Reduced hematocrit, hemoglobulin concentration and erythrocyte count. Increase in leukocyte count. Apparent hepatocellular necrosis and necrosis of the gastric glands. Dilation and changes in the profile of the head kidney. Increased hematopoietic activity of blood-forming tissues. Accumulation of iron pigments in the intestinal mucosal epithelium.	[93]
Aflatoxin B_1_	Red drum	*Sciaenops ocellatus*	0.1–5	7 weeks	Reduced weight gain and feed efficiency. Reduction in the whole-body lipid levels Liver lesions. Reduced survival.	[75]
Aflatoxin B_1_	Tilapia	*Oreochromis niloticus× O. aureus*	0.019–1.641	20 weeks	Reduced weight gain and growth. Yellowing of the body surface. Hepatic disorders—decrease in lipid content, infiltration by inflammatory cells and eosinophilic materials, white spots of necrosis, infiltration of macrophages and vacuolar degeneration of hepatocytes. Decrease in the concentration of total protein and albumin.	[78]
Aflatoxin B_1_	Nile tilapia	*Oreochromis niloticus*	0.9–3.0	25 days	Reduced growth feed intake. Histological changes in the liver—neoplastic changes and fatty liver. Congestion of the kidney, shrinking of the glomeruli and melanosis.	[79]
Aflatoxin B_1_	Nile tilapia	*Oreochromis niloticus*	0.25–100	8 weeks	Reduced weight gain. Reduced hematocrit. Histopathological changes in the liver—excess lipofuscin, irregularly sized hepatocellular nuclei and necrosis. Increased mortality.	[80]
Aflatoxin B_1_	Gibel carp	*Carassius auratus gibelio*	0.0032–0.9915	12–16 weeks	No significant health effects were determined. Low residues were found in muscles (<2.32 µg/kg).	[86]
Aflatoxin B_1_	Juvenile gibel carp	*Carassius auratus gibelio*	0.0032–0.0286	3 months	Slight damages to the hepatopancreas. Low levels of toxin in the muscle (<4.08 µg/kg).	[85]
Aflatoxins B_1_ + G_1_	Rainbow trout	Unspecified	0.0018–0.0397	Unspecified	Enlarged liver with histological changes—white or yellow nodules or cyst swellings, tumor-like lesions, irregular cords of abnormal hepatocytes, necrosis and hemorrhages. Enlarged hearts and kidneys. Ascites. Swollen abdomen.	[50]
Aflatoxins B_1_ + G_1_	Tilapia	Unspecified	0.0018–0.0397	Unspecified	No tumor-like lesions were detected.	[50]
Sterigmatocystin	Rainbow trout embryos	*Salmo gairdneri*	0.5	14 days	Increased incidence of hepatocellular carcinomas among survivors 1 year later.	[82]
Sterigmatocystin	Nile tilapia	*Oreochromis niloticus*	0.0016	4 weeks	Reduced body weight. Increase frequency of micronucleated red blood cells and chromosomal aberrations mainly in the kidney. Increased mortality. Accumulation (≈8 µg/kg) in the fish’s musculature.	[83]
Sterigmatocystin	Nile tilapia	*Oreochromis niloticus*	0.0016	4 weeks	Behavioral changes—unbalanced swimming. Darker color. Histopathological lesions in different organs—hyperplastic proliferation of bronchial epithelium, necrobiotic changes in hepatic and splenic tissues and destruction of components of the spleen. DNA changes—polymorphism band patterns.	[84]

**Table 2:** In vivo effects of fumonisin B_1_ on different fish species. Effects were dose-dependent.

Fish	Species	Dose (mg/kg)	Exposure Time	Effects	Ref.
Fry of channel catfish	*Ictalurus punctus*	20–40	10 weeks	Reduced weight gain and feed intake. Increase in feed conversion ratio. Reduction in hematocrit. Increased ratio of free sphinganine/free sphingosine in liver.	[98]
Channel catfish	*Ictalurus punctus*	0.3–720	10–14 weeks	Reduction in weight gain and feed intake. Lower hematocrit and red cell counts. Pale liver and kidneys. Liver lesions—white foci of subcapsular adipocyte hyperplasia, foci of swollen and shrunken hepatocytes and of hepatocellular necrosis. Yellow-white ventral portion of the liver. Higher mortality due to *Cytophaga columnaris* infection.	[97]
Channel catfish	*Ictalurus punctus*	35–313	5 weeks	No general negative health effects were noted. Mild enteritis.	[99]
Nile tilapia fingerlings	*Oreochromis niloticus*	10–150	8 weeks	Reduction in weight gain. Higher feed conversion rates. Lower hematocrit. Increase ratio between free sphinganine/free sphingosine.	[100]
Year-1 common carp	*Cyprinus carpio*	10 and 100	42 days	Reduction in body weight. Lesions in the liver, pancreas, kidney, heart, gallbladder and brain and damages in the blood vessels. Erythrodematitis lesions.	[101]

**Table 3:** In vivo effects of ochratoxin A in different fish species. Effects were dose dependent.

Fish	Species	Dose (mg/kg)	Exposure Time	Effects	Ref.
Sea bass	*Dicentrarchus labrax L.*	0.05–0.4	24–96 hours	Behavioral changes—sluggish movement, loss of equilibrium, rapid operculum movement, changes in the swimming pattern and respiratory manifestation. Muscular seizures prior to death. Hemorrhagic patches on the dorsal surface. Erosion of the fins and rusty spot formation in the belly region and dorsal musculature. General congestion of the kidney and gills. Spots of congestion on the periphery of the liver. Increased mortality.	[107]
Juvenile channel catfish	*Ictalurus punctus*	0.5–8.0	8 weeks	Reduction in body weight gain. Decrease in feed conversion rate. Reduction in hematocrit. Lesions in the liver and posterior kidney—increased incidence and severity of melanomacrophage centers in the hepatopancreatic tissue and posterior kidney and reduced number or absence of exocrine pancreatic cells surrounding the portal veins. Increased mortality.	[108]
Nile tilapia	*Oreochromis niloticus*	3% of fish bodyweight	14 days	Reduce weight gain, feed intake, final weight and feed conversion rate. Sluggish swimming Lesions in the liver, kidneys and spleen. Enlargement and congestion of kidney and liver. Dilation of blood vessels and necrosis of the kidney, degeneration and necrosis of hepatocytes. Pericarditis and myocarditis. Increased levels of alanine aminotransferase, aspartate transaminase, creatine and urea. Congested gills. Enlarged gallbladder. Reduction in total protein, albumin and globulin. Neutropenia. Higher mortality.	[110]
Atlantic salmon	*Salmo salar*	0.2–2.4	8 weeks	Increase in alkaline phosphatase, cholesterol, total protein, albumin and aspartate transaminase. Increased mRNA expression of immune marker in the spleen.	[117]

**Table 4:** In vivo effects of deoxynivalenol in different fish species. Effects were dose dependent.

Fish	Species	Dose (mg/kg)	Exposure Time	Effects	Ref.
Rainbow trout	*Oncorhynchus mykiss*	1.964	23 days	Gastrointestinal and liver hemorrhaging. Probable aneamia—lower values of erythrocyte haemoglobin, corpuscular haemoglobin concentration and haemoglobin in red blood. Probable decrease in the metabolism’s intensity—decrease in glucose, cholesterol and ammonia. Lesions in the caudal kidney—severe hyaline droplet.	[122]
Rainbow trout	*Oncorhynchus mykiss*	0.3–2.6	8 weeks	Reduction in weight gain and growth rate. Decrease in feed intake and feed efficiency. Decrease in recovered energy, energy retention efficiency, retained nitrogen and nitrogen retention efficiency. Lower whole-body crude protein concentration. Lesions in the liver—subcapsular hemorrhage and edema, fatty infiltration and altered hepatocytes.	[123]
Rainbow trout	*Salmo gairdneri*	0.001–0.013	4 weeks	Reduction in feed intake and feed conversion efficiency. Reduction in weight.	[124]
Rainbow trout	*Oncorhynchus mykiss*	3.3 and 6.4	4 weeks	Reduction in feed intake and weight gain. Increase in respiratory burst Reduction in susceptibility to *Flavobacterium psychrophilum* infection.	[125]
Atlantic salmon	*Salmo salar*	0.5–6	8 weeks	Reduced feed intake, feed efficiency, weight gain and length. Reduced packed cell volume. Decrease in concentration of alkaline phosphatase, cholesterol, triglycerides, total proteins and albumin. Reduced vaccination response against *Aeromonas salmonicidae*. Increased relative weight of the liver.	[120]

**Table 5:** In vivo effects of zearalenone in different fish species. Effects were dose dependent.

Fish	Species	Dose (mg/kg)	Exposure Time	Effects	Ref.
Zebrafish	*Danio rerio*	0.001	140 days (life-cycle)	Increased weight gain and body length on female fish. Feminization—shift towards female sex. Induction of plasma vitellogenin in females. Increased condition factor of the next generation. Decreased reproductive performance of the next generation. Does not affect fertility, hatch, embryo survival and gonad morphology.	[136]
Zebrafish	*Danio rerio*	0.1–3.2	42 days	Reduced of the relative spawning frequency. Reduced relative fecundity. Induction of plasma vitellogenin in male fish.	[135]
Rainbow trout	Unspecified	2	96 weeks (life cycle)	Increased feeding efficiency and growth rate. Modulation of the adaptative and innate immune system. Inflammation likely caused by pathogen infection. Changes in kidney morphology leading to atypical kidney structure and fibrosis—reddish spots displaying disorganized kidney morphology with inflammatory areas and granulomatous structures, whiteish spots or translucent, whiteish nodules. Rupture blood cells. Kidney inflammation was suggested to be due to *Tetracapsuloides bryosalmonae* infection. Increased albumin to globulin ratio (although not statistically significant). Decreased in lymphocytes concentration.	[137]
